# Relapse of nephrotic syndrome with unusual thromboembolic event: A case report

**DOI:** 10.1002/ccr3.7650

**Published:** 2023-08-16

**Authors:** Zahra Pournasiri, Seyedeh Masumeh Hashemi, Seyyedeh Narjes Ahmadizadeh, Bahareh Yaghmaei, Mitra Khalili, Azita Behzad, Amirali Soheili, Mahnaz Jamee

**Affiliations:** ^1^ Pediatric Nephrology Research Center, Research Institute for Children's Health Shahid Beheshti University of Medical Sciences Tehran Iran; ^2^ Pediatric Intensive Care department, Mofid Children's Hospital Shahid Beheshti University of Medical Sciences Tehran Iran; ^3^ Pediatric Department, Mofid Children's Hospital Shahid Beheshti University of Medical Sciences Tehran Iran; ^4^ Medical Student Research Committee, School of Medicine Shahid Beheshti University of Medical Sciences Tehran Iran

**Keywords:** coagulation, complications, nephrotic syndrome, thromboembolism

## Abstract

**Key Clinical Message:**

Most children with nephrotic syndrome heal without any sequelae. However, rare life‐threatening complications such as thromboembolism may occur in pediatric nephrotic syndrome and should be considered in those with a new‐onset neurologic deficit.

**Abstract:**

The thromboembolism (TE) as a complication of nephrotic syndrome (NS) is rare and serious, and may involve renal, cerebral, pulmonary, or peripheral venous and/or arterial thrombosis. Here, we describe a 4.5‐year‐old male with a history of nephrotic syndrome, who developed hemorrhagic stroke in the territory of middle cerebral artery (MCA).

## Graphical abstract

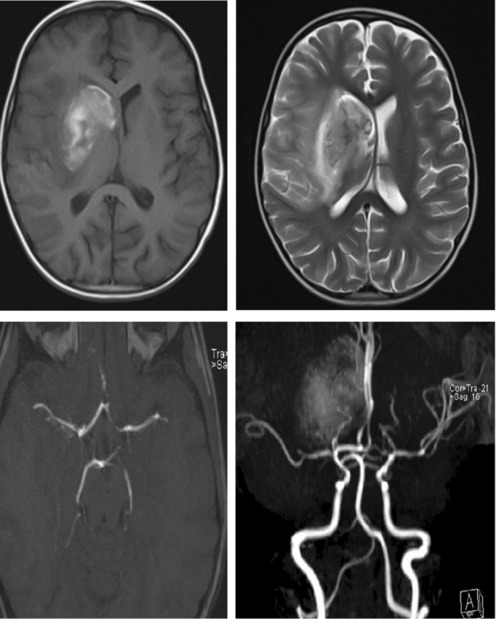

## 1 INTRODUCTION

Nephrotic syndrome (NS) is a renal disease classically characterized by proteinuria (urinary protein excretion greater than 40 mg/m^2^/h), hypoalbuminemia, edema, and hyperlipidemia.^1^ The estimated incidence of pediatric nephrotic syndrome is 1.9–4.7 per 100,000 children per year, varying by region and ethnicity.^2^ Various underlying etiologies can cause nephrotic syndrome, including congenital infection, glomerular disorders, vasculitis, viral and bacterial infections, toxins, malignancy, and genetic mutations. However, NS often happens in the absence of systemic disorders and the etiology is unknown in the majority of patients.^3^ NS is associated with heterogeneous complications including predisposition to infections (such as pneumonia, spontaneous bacterial peritonitis, urinary tract infection, acute gastroenteritis, empyema, etc.), generalized edema, bilateral pleural effusion, ascites, acute kidney injury, and thromboembolism (TE).^4^, ^5^, ^6^, ^7^, ^8^, ^9^, ^10^, ^11^ Most children with idiopathic nephrotic syndrome are steroid‐responsive, but relapse after the treatment of the first episode affects about 80% of patients with a median follow‐up of 60 months and more happen in children aged less than 4 years.^12^ Among aforesaid complications of NS in children, TE occurrence is significantly rare in comparison to its rate in adulthood. In this regard, we aim to describe the case of a 4‐year‐old male who presented with the relapse of nephrotic syndrome and two simultaneous life‐threatening complications, one related and another unrelated to his background disease.

## 2 CASE PRESENTATION

A 4.5‐year‐old male with a history of nephrotic syndrome was admitted to Mofid Children's Hospital with fever, abdominal pain, vomiting, and watery diarrhea in the last 2 days. He was born to closely related consanguineous parents. His sibling was deceased due to the brain structural abnormality at the first year of life.

Diagnosis of nephrotic syndrome was established 19 months earlier following the presentations of generalized edema and massive proteinuria. Work up for secondary nephrotic syndrome was turned out to be negative. Ultimately, with the diagnosis of minimal change nephrotic syndrome, he was treated with prednisolone and achieved remission quickly. He experienced two corticosteroid‐response relapses during this time, last one occurring 4 months ago.

At admission he was not receiving any mediation. In physical examination, he was a well‐developed and well‐nourished child with 105 cm height and 20 kg weight. The blood pressure was 110/80 mmHg and his temperature was 38°C. He was tachycardic and had respiratory distress. The respiratory rate was 45 per minute with 94% pulse oximetry. Moderate edema on bilateral eyelids and lower extremities, crackles in both lungs, and abdominal distention, tenderness and shifting dullness were also noted.

Laboratory findings revealed marked hypoalbuminemia (serum albumin: 1.2 g/dL) with massive proteinuria (protein/creatinine ratio: 19), hyperlipidemia (cholesterol: 176 mg/dL, triglyceride: 381 mg/dL), hypocalcemia (Ca: 5.9 mg/dL), hypophosphatemia (P: 4 mg/dL), with intact renal function (Cr: 0.6 mg/dL, BUN: 24 mg/dL).

Other laboratory results were as follows: Complete blood count: white blood cells (WBCs) = 15,000/mm^3^, neutrophils = 92%, lymphocytes = 6%, eosinophils = 1%, monocytes = 1%, hemoglobin = 12.6 g/dL, platelet = 325,000/μL, aspartate aminotransferase (AST) = 16 U/L, alanine aminotransferase (ALT) = 5 U/L, C‐reactive protein (CRP) = 27 mg; urinalysis: specific gravity = 1028, protein = 3+, glucose = trace, WBC = 6–8, red blood cells (RBCs) = many, blood = 3+, COVID‐19 polymerase chain reaction (PCR) = negative; peritoneal fluid analysis: glucose = 74 mg/dL, protein = 290 mg/dL, WBCs = 700/mm^3^, polymorph cells = 70%, lymphocytes = 30%, RBCs = 3000, lactate dehydrogenase (LDH) = 2031 U/L, albumin = 0.2 g/dL, amylase = 13 mg/dL, culture: negative.

There was no evidence of streptococcal infection. Based on his clinical and laboratory findings, his condition was considered as a combination of viral gastroenteritis, primary bacterial peritonitis, and nephrotic syndrome relapse. The patient was hospitalized and received cefotaxime, amikacin, and stress dose of hydrocortisone.

During admission abdomen gradually got more distended and guarding on palpation was recorded. Abdominal sonography revealed increased mural thickness of right and left colon and rectosigmoid and due to the severe ascites, appendix loop could not be evaluated. Kidneys were mildly enlarged and had increased parenchymal echogenicity.

In the abdominal laparotomy, acute inflammation and congestion of the serosal surface of the appendix and white fibrin around spleen and liver were detected and he underwent appendectomy.

**FIGURE 1:**
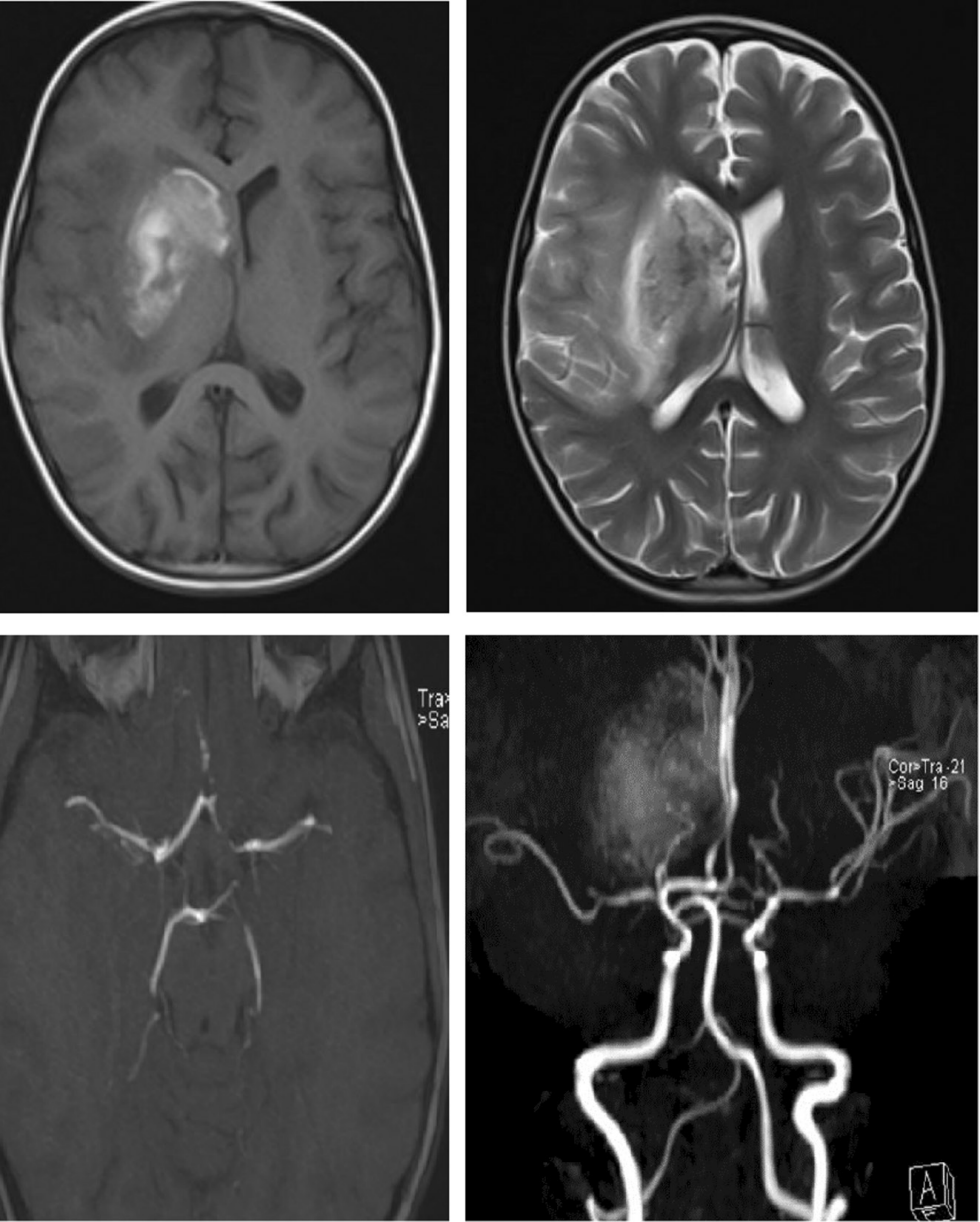
Magnetic resonance angiography (MRA), venography (MRV) and imaging (MRI) findings. Infarction in territory of middle cerebral artery (MCA) is noted with evidence of hemorrhage in right basal ganglia. In MRA filling defect in origin of M1 is noted, also with cutoff sign in the middle and superior branches of MCA at the level of M2. Findings are compatible with thrombosis in MCA and hemorrhagic infarct in the MCA territory.

After operation, his oxygen saturation dropped and required mechanical ventilation. During intensive care unit (ICU) admission, he developed pneumonia and pulmonary edema, massive pleural effusion, and congestive heart failure. Hence, antibiotic therapy with vancomycin and meropenem started and after 2 weeks he got extubated. However, 2 days later, he developed left‐sided flaccid hemiplegia. The brain magnetic resonance angiography (MRA) showed hypodensity in the territory of middle cerebral artery (MCA) in the right side with hemorrhagic transformation, indicating hemorrhagic stroke (Figure 1).

Consequently, a continuous unfractionated heparin infusion was started. No prior history of thrombotic event was reported and hypercoagulable labs were unrevealing (Table 1).

**TABLE 1:** Coagulation profile of the index patient.

Test name	Result	Unit	Reference range
Factor VIII	210	%	48–124
Factor XIII (screen)	Normal		Normal
Fibrinogen	360	mg%	188–413
Protein C	160	%	59–147
Protein S	60	%	60–140
Antithrombin III	135	%	61–128
Lupus anticoagulant	Neg		
DRVV screen	48	s	32–46
DRVV confirm	35	s	31–40
DRVV ratio	1.2		0.3–1.2
PTT LA screen	44	s	31–47

The patient remitted after 2 weeks with stress dose of corticosteroid. His renal function remained in the normal range with no further complication.

After clinical resolution, the patient was discharged with prednisolone (40 mg/m^2^, every other day), enoxaparin, vitamins, and calcium supplement. Also, he was recommended for pneumococcal vaccination. One year after discharge, the patient was in remission and the hemiplegia was considerably improved. He can walk alone but has some restriction with hand function.

## 3 DISCUSSION

Infection and thromboembolic event are two main complications of nephrotic syndrome.^10^ We reported an uncommon case of NS associated with both of these serious complications and simultaneous appendicitis as an unrelated event.

The absolute risk of venous and arterial thrombosis are eight times higher than that observed in general population and usually occurs within the first 6 months of diagnosis.^13^, ^14^, ^15^ The incidence of TE in children with NS is much lower (1.8%–5.3%) than in adults (26.7%).^16^

A systematic review by Stabouli et al.^6^ showed that the most common neurological complications in pediatric nephrotic syndrome are cerebral TE and posterior reversible encephalopathy syndrome (PRES). Less common complications include Wernicke's encephalopathy, diffuse cerebral hypoperfusion, and Guillain–Barre syndrome.^17^, ^18^, ^19^ Cerebral TE mainly manifests at the age of 7.2 years, with a male predominance (70.1%). 71.6% of cerebral TEs occur during disease relapse, mostly in the setting of focal segmental glomerulosclerosis (FSGS**).** ^6^

The index patient had at least five risk factors contributing to his thrombotic event, including severe hypoalbuminemia, bacterial infection, central venous line insertion, severe heart failure, and abdominal surgery. Other known TE‐associated risk factors include immobility, hemoconcentration, use of diuretics and steroids, orthopedic surgery, and genetic thrombophilic tendency.^20^ Although the association of TE and NS has been recognized since 1948, the pathophysiology and management need to be elucidated. It is thought to be caused by an imbalance of procoagulant and anticoagulant factors such as increased levels of procoagulant proteins (fibrinogen, Factors V and VIII), increased platelet activation, and decreased levels of natural anticoagulants like antithrombin III, plasminogen, and protein C and S (due to urinary losses).^21^

In the NS, every vein may be somewhere affected by thrombosis, although foremost frequent sites are the vessels of the lower extremities, followed by rare involvement of cerebral venous plexus, renal vein, inferior vena cava, and hepatic vein.^22^, ^23^, ^24^ In addition, arterial thrombosis has been reported in nephrotic patients in renal, pulmonary, coronary, and cerebral arteries, and less frequently in the aorta, mesenteric, axillary, subclavian, brachial, femoral, ophthalmic, and carotid arteries.^25^ Igarashi et al. reported a 11‐year‐old male with the diagnosis of NS presenting with right frontal headache, confusion, and right‐sided weakness. At admission, imaging studies (CT‐scan) did not show any sign of infraction or hemorrhage; however, 5 days later large area of infarction in the left MCA was detected by CT‐scan.^26^

Acute cerebral hemorrhage in NS is extremely rare. Mengqi Yang et al. reported 15 adult cases of cerebral hemorrhage among 10,461 patients with NS during 10 years.^27^ Cerebral hemorrhage in pediatric nephrotic syndrome is even more rare and are reported in few cases.^5^, ^28^, ^29^ Hu et al reported an 8‐year‐old girl who presented with unconsciousness and relapse of nephrotic syndrome. Her brain CT scan showed spontaneous intracerebral hemorrhage within the left occipital lobe.^5^ Another case was a 12‐year‐old boy with nephrotic syndrome and generalized convulsions duo to a subcortical hematoma in the left frontal lobe.^28^ Erbenand et al. reported a 8‐year‐old patient with dorsalis pedis artery and posterior tibial artery thrombosis during relapse of nephrotic syndrome which eventually led to the distal digital amputation.^29^

Shannon L. Carpenter et al. showed association of longer hospitalizations, ICU stays, and infections with venous TE (VTE) in children with nephrotic syndrome. This study on 370 hospitalized children showed a median ICU admission of 4 days in those patients with VTE compared to 0 days in those without VTE. In addition, patients with VTE were younger and had higher rates of infections.^30^ This study was compatible with our patient who had more than 1 month admission in ICU and several infectious diseases including gastroenteritis, peritonitis, and pneumonia.

Another interesting point in our case was his quick remission with no further complications in the follow up, without administration of classic full dose of corticosteroid. In the same manner, a prospective observational study on 45 children with steroid‐sensitive NS, presented an infection‐associated relapse, 60% of those patients achieved remission with treatment of infection with/without the use of stress doses of prednisolone, and the majority were still in remission at 3 months follow‐up.^31^

Most NS cases heal without any complications, but in some cases several complications might occur. Therefore, life‐threatening consequences such as thromboembolic events should be considered in patients with NS and these patients should be observed by different specialists and evaluated for risk factors of this complications.

## AUTHOR CONTRIBUTIONS

**Zahra Pournasiri:** Conceptualization. **Seyedeh Masumeh Hashemi:** Data curation. **Seyyedeh Narjes Ahmadizadeh:** Visualization. **Bahareh Yaghmaei:** Writing – original draft. **Mitra Khalili:** Validation. **Azita Behzad:** Supervision. **Amirali Soheili:** Writing – original draft. **Mahnaz Jamee:** Writing – review and editing.

## FUNDING INFORMATION

This research received no specific grant from any funding agency in the public, commercial, or not‐for‐profit sectors.

## CONFLICT OF INTEREST STATEMENT

The authors declare that they have no conflict of interest.

## CONSENT

Written informed consent was obtained from the patient's parents to publish this report in accordance with the journal's patient consent policy.

## Data Availability

Data sharing is not applicable to this article as no new data were created or analyzed in this study.
